# Distinct common signatures of gut microbiota associated with damp-heat syndrome in patients with different chronic liver diseases

**DOI:** 10.3389/fphar.2022.1027628

**Published:** 2022-11-17

**Authors:** Yuqing Pan, Jianchun Guo, Na Hu, Yunhao Xun, Binbin Zhang, Qin Feng, Si Chen, Xiaojing Li, Qiaohong Liu, Yiyang Hu, Yu Zhao

**Affiliations:** ^1^ Key Laboratory of Liver and Kidney Diseases (Ministry of Education), Shanghai Key Laboratory of Traditional Chinese Clinical Medicine, Institute of Liver Diseases, Shuguang Hospital Affiliated to Shanghai University of Traditional Chinese Medicine, Shanghai, China; ^2^ Department of Integrative Medicine, Hangzhou Xixi Hospital, Hangzhou, China; ^3^ Institute of Clinical Pharmacology, Shuguang Hospital Affiliated to Shanghai University of Traditional Chinese Medicine, Shanghai, China

**Keywords:** traditional Chinese medicine, damp-heat syndrome, gut microbiota, chronic hepatitis B, non-alcoholic fatty liver disease

## Abstract

**Background:** Chronic hepatitis B (CHB) and non-alcoholic fatty liver disease (NAFLD) are prevalent in China. According to traditional Chinese medicine (TCM) theory, damp-heat (DH) syndrome is common in chronic liver disease. However, the biological characteristics related to quantitative diagnosis remain to be determined. This study aimed to identify the consistent alterations in the gut microbiota associated with DH syndrome in patients with CHB or NAFLD.

**Methods:** A total of 405 individuals were recruited, of which 146 were participants who met the consistent TCM diagnosis by three senior TCM physicians and were typical syndromes. All participants were required to provide fresh stool and serum samples. The gut microbiota was assessed by fecal 16S rRNA gene sequencing, and the serum metabolite profiles of participants were quantified by an ultra-performance liquid chromatography coupled to tandem mass spectrometry (UPLC-MS/MS) system. DH syndrome-related bacteria taxa were identified based on the 146 individuals with typical syndromes and validated in all 405 volunteers.

**Results:** The results showed that CHB and NAFLD patients with typical TCM DH syndrome had consistently elevated serum total bile acid (TBA) levels. Significant alterations in microbial community were observed according to TCM syndromes identification. A total of 870 microbial operational taxonomic units and 21 serum metabolites showed the same variation trends in both the CHB and NAFLD DH syndrome groups. The functional analysis predicts consistent dysregulation of bile acid metabolism. Five genera (*Agathobacter, Dorea, Lachnospiraceae_NC2004_group, Subdoligranulum,* and *unclassified_c__Clostridia*) significantly decreased in abundance in patients with DH syndrome. We utilize these five genera combined with TBA to construct a random forest classifier model to predict TCM diagnosis. The diagnostic receiver-operator characteristic (ROC) areas for DH syndrome were 0.818 and 0.791 in internal tenfold cross-validation and the test set based on all 405 individuals, respectively.

**Conclusion:** There are common signatures of gut microbiota associated with DH syndrome in patients with different chronic liver diseases. Serum TBA combined with DH-related genera provides a good diagnostic potential for DH syndrome in chronic liver disease.

## Introduction

Chronic hepatitis B (CHB) and non-alcoholic fatty liver disease (NAFLD) are currently the most prevalent forms of chronic hepatitis in China (Wang et al., 2014; Zhou et al., 2020). Traditional Chinese medicine (TCM) is widely used in China, as well as in the treatment of chronic liver disease. TCM is advanced at alleviating clinical symptoms and improving liver function in patients with liver diseases (Zhang et al., 2010). TCM prescription based on *Zheng* pattern identification. “*Zheng*”, also known as TCM syndrome, is a term that generalizes the pathological properties and changes associated with certain stages of TCM disease. The TCM practitioner identifies the patient’s syndrome diagnosis by a comprehensive symptom-based approach according to observation, auscultation, inquiry, and palpation. However, the symptoms that TCM syndrome pattern diagnosis relies on are subtle and unquantifiable (such as fatigue and thirst). TCM diagnosis may differ depending on the subjective observations and clinical experience of the TCM practitioner. The same condition of a patient may be diagnosed differently by different TCM clinicians, the inconsistency hinders the repeatability of TCM, and even spark a potential argument about TCM as a medical system. Therefore, it is essential to explore the quantitative and valid biological scientific foundation of TCM syndromes to enhance TCM diagnosis and modernization (Jiang et al., 2012).

Damp-heat (DH) syndrome is a status of disharmony that often occurs when dampness binds with heat evil. DH syndrome can be seen in a variety of modern medical diseases and is a common syndrome pattern in chronic liver disease. This pattern often results from the internal accumulation of damp heat and is characterized by distending pain/masses in the subcostal region, thirst, and a bitter mouth. The tongue is red with a yellow, greasy coating. The pulse is slippery and rapid. DH syndrome is very common in patients with chronic liver diseases, which is accounting for 21.9% of NAFLD patients (Li et al., 2019; Zhou et al., 2022). According to a systematic review based on 20,106 participants, DH occurs in 32.2% of all patients with CHB (Zeng et al., 2011). A crucial concept in TCM theory is that “different diseases have the same syndrome”. CHB is a liver infection caused by persistent hepatitis B virus (HBV) (Seto et al., 2018), and NAFLD is characterized by excessive fat accumulation in the hepatocytes (Loomba et al., 2021). However, the prescriptions against DH syndrome may be similar for patients with either CHB or NAFLD in TCM. For example, *Yinchenhao* decoction is a classic Chinese medicine prescription, widely used in various liver diseases accompanied by dampness-heat syndrome and jaundice. Therefore, DH syndrome may share a common biological basis in different liver diseases.

In recent years, multi-omics have linked TCM and biomolecular changes. Our previous study showed that CHB and NAFLD patients with DH syndrome have certain common urinary metabolic disorders (Dai et al., 2013). According to TCM theory, DH syndrome reflects systemic changes in the entire body but mainly indicates dysfunction in digestive tract organs, such as the stomach, intestines, and liver. The human gastrointestinal system contains trillions of microorganisms. Gut microbiota strongly influences host homeostasis (Fan and Pedersen, 2021). Certain TCM herbal formulations and compounds are found to clear damp heat evil by modulating gut microbiota (Wu et al., 2020; Xu et al., 2020). However, the relationship between the gut microbiota and DH syndrome in patients with chronic liver diseases remains to be determined. In the present study, we investigated the distinct signatures common to CHB and NAFLD associated with DH syndrome on the basis of patient intestinal microbiota and serum metabolites.

## Methods

### Patient recruitment

From July 2019 to November 2020, patients with CHB or NAFLD who met the established inclusion and exclusion criteria were enrolled in this study. Healthy volunteers were enrolled as healthy control. Volunteers in this study originated from Shuguang Hospital affiliated with Shanghai University of Traditional Chinese Medicine (Shanghai, China), Shanxi Provincial Hospital of Traditional Chinese medicine (Taiyuan, China), and Hangzhou Xixi Hospital (Hangzhou, China). All participants were required to provide fresh stool and serum samples and basic information, be tested for disease-related laboratory indices and fill out TCM syndrome and diet questionnaires. The present study was conducted following the Declaration of Helsinki and was approved by the institutional review board (IRB) of Shuguang Hospital (No. 2019-662-17-01).

CHB was diagnosed based on the criteria of “Chinese guidelines on prevention and treatment of chronic hepatitis B” (2015 version) (GuiQiang, 2015). NAFLD was diagnosed according to the “Chinese guidelines on diagnosis and treatment of non-alcoholic fatty liver disease” (2010 version) (Fan Jiangao, 2010). Briefly, CHB diagnosis comprised a thorough examination and routine laboratory tests including liver function, hepatitis B virus (HBV) replication markers, and abdominal ultrasound. NAFLD diagnosis required confirmation of significant hepatic steatosis by imaging or histology and the absence of any secondary causes of hepatic steatosis.

Eligible patients with chronic liver diseases included those who were diagnosed with CHB or NAFLD, were 18–65 years of age, and signed an informed consent form. Those who were diagnosed with other chronic liver diseases such as other viral hepatitis, alcoholic fatty liver disease, cirrhosis, liver cancer, and NAFLD co-infected with HBV were excluded. Those with serious primary disease or malignant tumor, who had undergone gastrointestinal bariatric surgery or taken weight loss drugs within the past 3 months, who had taken antibiotics or proton pump inhibitors within the past month, or were pregnant or lactating were also excluded. Healthy controls were defined as individuals with normal routine laboratory tests, no diagnosis of chronic liver disease, and no abnormal TCM symptoms.

### TCM syndrome diagnosis

**FIGURE 1:**
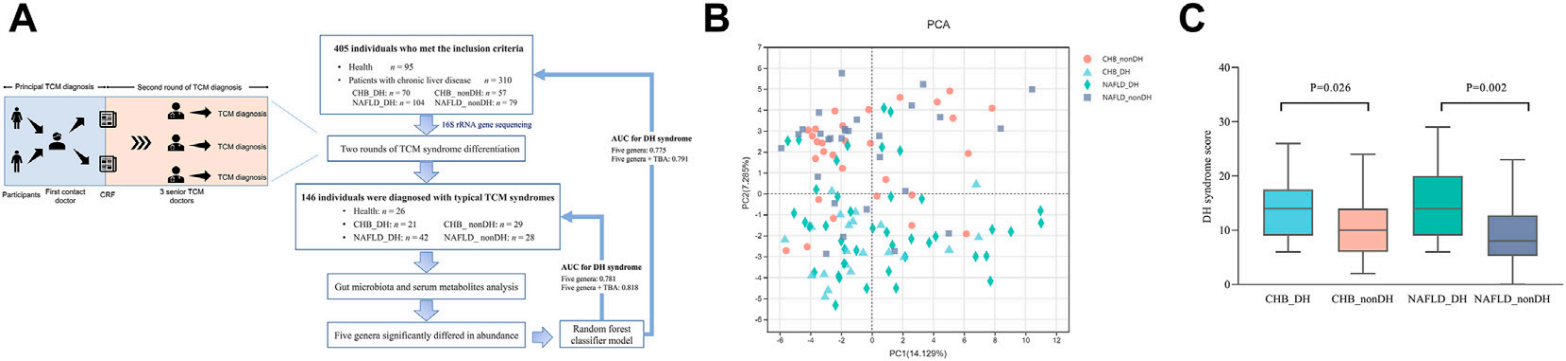
Analysis of TCM information for 146 individuals. **(A)** Flowchart of this study; **(B)** PCA analysis based on TCM diagnostic parameters; **(C)** DH syndrome score comparison.

TCM syndrome was diagnosed by “Guidelines for clinical research on new drugs of traditional Chinese medicine” (Xiaoyu, 2002). We performed two independent rounds of syndrome pattern identification to ensure the quality. As seen in Figure 1A, the first contact doctor received the participant, collected the participant’s TCM symptom information, took tongue images, recorded in the case report form (CRF), and gave the principal TCM diagnosis in the CRF based on the information obtained at the consultation. The second round of diagnosis was conducted by three senior doctors practicing TCM for over 30 years independently according to the TCM symptom information recorded in the CRF.

The CRF contained 55 items of conventional TCM symptoms derived from the four diagnostic methods. Most symptom is divided into grades of none, mild, general, severe, and intolerable according to the frequency or degree. Each pulse item was recorded as present or absent. The TCM syndrome score of DH syndrome is based on the combination and degree of DH symptom-related positive items by the “Guidelines for clinical research on new drugs of traditional Chinese medicine” (Xiaoyu, 2002). For the different degrees of each item (none, mild, general, severe, and intolerable), the main symptoms were assigned a score of 0, 2, 4, 6, and eight; corresponding to secondary symptoms of 0, 1, 2, 3, 4, respectively.

### Diet history questionnaire

Since diet has a considerable effect on intestinal microecology, we required all participants to fill out a dietary questionnaire, which accounts for the consumption of any seafood, tea, or wine during the day before stool collection. We also inquired about the forms, regularity, and colors of stool in the questionnaire.

#### Stool sample collection, fecal 16S rRNA sequencing and data processing

Before fecal sample collection, volunteers were required to avoid the consumption of drugs, seafood, strong tea, alcohol, and any other foods that significantly influence gut microbiota. Researchers distributed the stool collection kits before enrollment and informed the subjects of the sampling process and precautions. Subjects collected fecal samples between 20:00 on the night before enrollment and the morning of enrollment. The samples were placed in transport boxes with ice packs. Upon receipt, the samples were immediately frozen at −80°C.

The following steps were performed at Majorbio Bio-Pharm Technology Co. Ltd. (Shanghai, China). Total microbial community DNA was extracted according to the instructions of an E.Z.N.A.^®^ Soil DNA kit (Omega Bio-tek, Norcross, GA, United States). DNA extract quality was determined using 1% agarose gel electrophoresis. DNA concentration and purity were determined with a NanoDrop2000 spectrophotometer (Thermo Fisher Scientific, Waltham, MA, United States). PCR amplification of the V3-V4 region of the 16S rRNA gene was performed using the primers 338F (5ʹ-ACT​CCT​ACG​GGA​GGC​AGC​AG-3ʹ) and 806R (5ʹ-GGACTACHVGGGTTWTCTAAT-3ʹ). The PCR products from the same sample were pooled, recovered with 2% agarose gel, purified with an AxyPrep DNA Gel Extraction Kit (Axygen Biosciences, Union City, CA, United States), and detected using 2% agarose gel electrophoresis. The recovered product was detected and quantified with a Quantus™ fluorometer (Promega, Madison, WI, United States). The libraries were constructed with a NEXTFLEX^®^ Rapid DNA-Seq kit (PerkinElmer, Waltham, MA, and United States). Sequencing was performed on the Illumina MiSeq platform (Shanghai Majorbio Bio-Pharm, Shanghai, and China). Fastp (https://github.com/OpenGene/fastp) was used for quality control of the original sequence and FLASH (http://ccb.jhu.edu/software/FLASH/) was used for splicing. Uparse v. 7.0.1090 (http://drive5.com/uparse/) was used to cluster the sequences by operational taxonomic unit (OTU) and according to a 97% similarity threshold. Chimeras were eliminated.

Data processing was performed at Majorbio Bio-Pharm Technology Co. Ltd. (Shanghai and China; https://cloud.majorbio.com). Based on the Silva 16S rRNA database (v. 138; https://www.arb-silva.de), the RDP classifier Bayesian algorithm (https://sourceforge.net/projects/rdp-classifier/) was used to annotate the representative OTU sequences with 97% similarity. A confidence threshold of 0.7 was set to obtain the species taxonomy annotation results. The alpha diversity index of the intestinal microbiota was analyzed using mothur (v. 1.30.2; https://mothur.org/wiki/calculators/) (Schloss et al., 2009). Principal co-ordinate analysis (PCoA) and partial least squares discriminant analysis (PLS_DA) were used to compare OTU-level gut microbiota compositions. Significantly altered bacteria and metabolites were identified by Wilcoxon’s rank-sum test at a significance level of *p* < 0.05. Biomarker’s taxa were identified with Linear discriminant analysis (LDA) effect size (LEfSe) using the Majorbio online platform (Shanghai and China; https://cloud.majorbio.com). PICRUST2 (https://github.com/picrust/picrust2) was used for functional predictions. Multivariate association analysis was performed using the MaAsLin2 R package to identify specific taxa associated with the host phenotype without the influence of other metadata. We adjusted for age, sex, and BMI as fixed variables. *p* < 0.05 were considered significant. We built Gaussian mixture models using MATLAB (v. 9.0.0) (The MathWorks Inc., Natick, MA, and United States). To evaluate the potential diagnostic ability of DH-associated bacterial genera for DH syndrome, we constructed random forest models and yielded receiver operating characteristic (ROC) curves using Weka (v. 3.8.6) (Machine Learning Group, University of Waikato, Hamilton, and NZ). Raw data were uploaded to the National Center for Biotechnology Information (NCBI) under Sequence Read Archive (SRA) No. SRP361117 (https://www.ncbi.nlm.nih.gov/sra/?term=PRJNA809132).

#### Serum metabolite analysis

Metabolomics analysis was performed using a Q300 Kit (Metabo-Profile, Shanghai, and China). All target metabolites were quantitatively analyzed using ultraperformance liquid chromatography coupled to tandem mass spectrometry (UPLC/MS) (ACQUITY UPLC-Xevo TQ-S, Waters Corp., Milford, MA, United States, and Metabo-Profile Biotechnology (Shanghai) Co. Ltd). Blood samples were collected from the peripheral veins after the volunteers fasted for 8 h. The blood was centrifuged at 3,500 × *g* and 4°C for 15 min and the supernatant was collected and immediately stored at −80°C. The samples were thawed on an ice bath to minimize degradation. Serum (20 μl) was added to each well of a 96-well plate. The plate was transferred to an Eppendorf epMotion Workstation (Eppendorf GmbH, Hamburg, and Germany). Then, 120 μl ice-cold methanol including partial internal standards was automatically added to each sample and the mixtures were vortexed for 5 min. The plate was centrifuged (Allegra X15R, Beckman Coulter, Inc., Indianapolis, IN, and United States) at 4,000 × *g* for 30 min and returned to the workstation. Then 30-μl supernatant was transferred to a clean 96-well plate and 20 μl of freshly prepared derivatizing reagent mixture was added to each well. The plate was sealed and derivatized at 30°C for 60 min. Then 330 μl ice-cold 50% (v/v) methanol solution was added to each well. The plate was then stored at −20°C for 20 min and centrifuged at 4,000 × *g* and 4°C for 30 min. Then 135 μl of supernatant was transferred to each well of a fresh 96-well plate. Each well contained 10 μl of internal standards. Serial dilutions of the derivatized stock standards were added to the left-side wells. Raw data files generated by UPLC-MS/MS were processed using MassLynx v.4.1 (Waters Corp.) for peak integration, calibration, and quantitation of each metabolite. The plate was then sealed for the LC-MS analysis. All targeted metabolite standards were obtained from Sigma-Aldrich (St. Louis, MO, and United States), Steraloids Inc. (Newport, RI, United States), and TRC Chemicals (Toronto, ON, Canada).

The data were analyzed with iMAP v. 1.0 (Metabo-Profile). Partial least-squares discriminant analysis (PLS_DA) was established to visualize differences in metabolite profiles. Metabolite enrichment analysis was performed on Metaboanalyst v. 5.0 (https://www.metaboanalyst.ca/). |Fold change (FC)| > 1.2 and p values in Wilcoxon’s rank-sum test were used to estimate the significance of each metabolite.

#### Statistics

Clinical data was expressed as means ± standard deviation, median (interquartile range), or number (%) depending on the distribution. Student’s *t*-test, Mann-Whitney *U* test, or Chi-square test was used to analyze significant differences between groups using SPSS (v. 26.0) (IBM Corp., Armonk, NY, and United States). A p-value less than 0.05 was considered statistically significant. The Spearman’s rank or Pearson’s correlation test was used for correlation analysis using Origin (v.9.8.0.200) (OriginLab, Northampton, MA, and United States). Images were plotted using GraphPad Prism (v. 9.0.0) (GraphPad Software, La Jolla, CA, and United States) or R (v. 4.2.0) (R Core team, Vienna, and Austria).

## Results

### Serum total bile acid expression increased in CHB and NAFLD patients with DH syndrome

The present study included 405 volunteers (95 healthy controls, and 310 patients with chronic liver disease). After two rounds of syndrome differentiation, 146 participants were diagnosed consistently with typical TCM syndrome. Among them, 26 were healthy volunteers (H group) and 120 were patients diagnosed with CHB (41.7%) and NAFLD (58.3%). We combined the other syndromes commonly observed in chronic liver diseases, such as spleen deficiency, liver-kidney Yin deficiency, and blood stasis syndromes, into the non_DH syndrome group. We diagnosed 21 CHB and 42 NAFLD volunteers with typical DH syndrome (CHB_DH and NAFLD_DH, respectively); and 29 CHB and 28 NAFLD patients without DH syndrome were identified as the non_DH syndrome groups (CHB_nonDH and NAFLD_nonDH, respectively) (Figure 1A).

There was no significant difference between patients with and without DH syndrome in terms of their basic information (*p* > 0.05; Table 1). A principal component analysis (PCA) based on TCM symptom data showed patients with chronic liver diseases with DH syndrome had a separation trend from the non_DH groups (Figure 1B). The DH syndrome scores based on TCM symptoms also differed significantly between patients with and without DH syndrome (*p* < 0.05; Figure 1C). Compared with the CHB_nonDH group, the patients with CHB DH syndrome exhibited elevated serum aspartate aminotransferase (AST), alkaline phosphatase (ALP), TBA, and FibroScan liver stiffness measurement (LSM) scores but decreased serum albumin (ALB) (*p* < 0.05). The trends for these clinical markers in NAFLD-DH patients were consistent, especially the serum TBA exhibited a consistent statistical difference between patients with and without DH syndrome (*p* < 0.05) (Table 1).

**TABLE 1:** The basic information of participants.

Characteristic	H (*n* = 26)	CHB_DH (*n* = 21)	CHB_nonDH (*n* = 29)	Statistical value	*p*-value*	NAFLD_DH (*n* = 42)	NAFLD_nonDH (*n* = 28)	Statistical value	*p*-value^#^
Gender (Male/Female)	10/16	16/5	17/12	0.851^a^	0.196	30/12	17/11	0.711^a^	0.350
Age (years)	29.69 ± 5.01	41.04 ± 12.63	38.34 ± 9.81	1.676^b^	0.399	44.61 ± 12.78	42.46 ± 11.83	0.874^b^	0.479
BMI (kg/m^2^)	21.65 ± 1.74	22.32 (5.16)	21.14 (5.53)	−1.032^c^	0.302	27.42 (4.20)	26.25 ± 2.94	−1.547^c^	0.121
TBiL (μmol/L)	15.17 ± 5.41	14.50 (10.55)	12.60 (8.90)	−0.748^c^	0.188	12.70 (7.38)	13.33 (3.28)	1.041^a^	0.701
DBiL (μmol/L)	3.4 (2.38)	4.95 (4.18)	4.30 (2.75)	−0.248^a^	0.107	3.25 (1.65)	2.94 (1.03)	0.414^a^	0.388
ALT (U/L)	12.1 (9.38)	28.80 (148.35)	19.80 (13.65)	0.548^a^	0.014	30.15 (39.83)	28.80 (25.94)	−1.273^a^	0.976
AST (U/L)	17 (7.5)	28.80 (53.75)	22.70 (10.50)	−1.317^c^	0.007	24.55 (14.55)	25.45 (14.33)	−0.384^c^	0.824
GGT (U/L)	15.3 (9.21)	28.80 (71.24)	17.37 (10.69)	−1.612^c^	0.017	33.54 (27.14)	34.41 (25.54)	−0.864^c^	0.769
ALP (U/L)	65.48 ± 19.04	105 (45.35)	83.27 ± 21.35	−2.447^c^	0.026	85.27 ± 21.97	86.68 ± 12.37	−0.030^c^	0.733
ALB (g/L)	44.91 ± 2.86	42.04 ± 4.22	45.38 ± 3.69	−2.713^c^	0.005	44.79 ± 3.63	45.52 ± 2.33	−0.222^c^	0.315
TBA (μmol/L)	1.30 (0.99)	3.20 (6.65)	1.0 (1.15)	−2.389^c^	0.001	1.60 (1.23)	0.80 (1.16)	−0.294^c^	0.006
FBG (mmol/L)	4.78 ± 0.33	4.55 ± 0.79	4.81 ± 0.42	−2.231^c^	0.176	4.97 (1.53)	5.28 (1.02)	−0.342^a^	0.557
HDL (mmol/L)	1.36 ± 0.24	1.19 ± 0.25	1.31 ± 0.34	−2.971^a^	0.193	1.00 (0.31)	1.05 ± 0.22	−1.013^a^	0.746
LDL (mmol/L)	2.64 ± 0.57	2.52 ± 0.47	2.65 ± 0.65	−3.474^c^	0.435	3.10 ± 0.75	3.29 ± 0.64	−2.754^c^	0.291
TC (mmol/L)	4.52 ± 0.69	4.37 ± 0.69	4.33 ± 0.91	−1.387^a^	0.896	4.92 ± 0.90	5.18 ± 0.87	−0.588^c^	0.233
TG (mmol/L)	0.82 (0.52)	1.09 (0.50)	0.91 (0.66)	−1.321^a^	0.157	2.12 (1.57)	1.64 (1.56)	−0.324^c^	0.479
LSM score (kPa)	5.13 (1.15)	7.85 (2.23)	5.50 (1.55)	−0.787^a^	0.004	5.99 ± 1.73	5.60 (2.43)	−1.063^a^	0.353
CAP score (db/m)	224.0 (16.75)	217.36 (8.23)	210.21 ± 33.00	0.132^a^	0.192	301.21 ± 35.60	295.00 (31.75)	−1.202^a^	0.359

*p-value denotes differences between CHB_DH, and CHB_nonDH.

^#^ *p*-value denotes differences between NAFLD_DH, and NAFLD_nonDH.

^a^ Student’s *t* test.

^b^ Chi-square *χ* ^*2*^ test.

^c^ Mann-Whitney *U* test. Abbreviations: TBiL, total bilirubin; DBiL, direct bilirubin; ALT, alanine aminotransferase; AST, aspartate aminotransferase; GGT, gamma-glutamyl transferase; ALP, alkaline phosphatase; ALB, albumin; TBA, total bile acid; FBG, fasting blood glucose; TC, total cholesterol; TG, triglycerides; HDL-C, high-density lipoprotein cholesterol; LDL-C, low-density lipoprotein cholesterol; LSM, hepatic fibrosis index-liver stiffness measurement; CAP, hepatic steatosis index-controlled attenuation parameter.

### Patients with DH syndrome are accompanied by significant alterations in the gut microbial community

**FIGURE 2:**
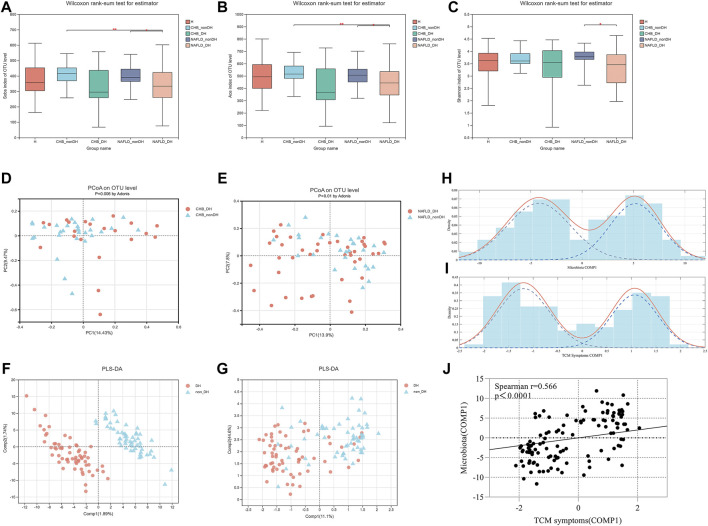
Comparison of α diversity and β diversity of gut microbial communities in patients with and without DH syndrome. **(A)** Sobs index; **(B)** Ace index; **(C)** Shannon index; PCoA with Bray-Curtis distance at OTU level based on **(D)** CHB_DH and CHB_nonDH individuals and **(E)** NAFLD_DH and NAFLD_nonDH individuals; **(F)** Score plot of PLS_DA analysis based on gut microbiota OTUs in patients with and without DH syndrome (the R^2^Y and Q^2^ values for the model were 0.988 and 0.469, respectively); **(G)** Score plot of PLS_DA analysis based on TCM symptoms in patients with and without DH syndrome (the R^2^Y and Q^2^ values for the model were 0.448 and 0.275, respectively); Finite Gaussian mixture models of parameters of **(H)** COMP1 in Figures 2F, **(I)** COMP1 in Figure 2G; **(J)** Spearman’s correlation analysis of both COMP1 parameters.

There were no differences in seafood, tea, wine consumption between groups (Supplementary Table S1). A total of 19,601,356 high-quality sequences (48,398.4 ± 9,426.31 reads) and 2,156 operational taxonomic units (OTUs) from 405 samples were obtained. The value of the good’s coverage estimator showed that the sequencing depth was enough (Supplementary Figure S1A). Alpha-diversity based on the fecal samples of 146 participants showed that compared with the non_DH syndrome group, the CHB_DH and NAFLD_DH groups had lower gut microbiota abundance and diversity. The Sobs, Ace, and Shannon indices significantly differed between the NAFLD_DH and NAFLD_nonDH groups (*p* < 0.05) (Figures 2A–C). The trends in the whole 405 individuals were the same (Supplementary Figures S1B–D).

An OTU-level PCoA based on the Bray-Curtis dissimilarity index showed that the gut microbiota community compositions significantly differed between the patients in CHB_DH and CHB_nonDH (*p* = 0.006) and between those in NAFLD_DH and NAFLD_nonDH groups (*p* = 0.01) (Figures 2D,E). To clarify the correlation between the gut microbiota and the TCM syndromes, we combined CHB_DH and NAFLD_DH groups into the DH syndrome group and the patients without DH syndrome into the non_DH group. The PLS_DA analysis based on the gut microbiota OTUs disclosed that the DH syndrome and non_DH syndrome groups separated in the first component (COMP1) (Figure 2F). The PLS_DA analysis based on the TCM symptoms also revealed a separation trend between the DH syndrome and non_DH syndrome groups in COMP1 (Figures 2F,G). We built finite Gaussian mixture models to fit the COMP1 parameters in Figures 2F,G, respectively. They conformed to two normal distributions corresponding to the TCM syndrome groupings (Figures 2H,I). Both COMP1 parameters were significantly correlated (*p* < 0.001) (Figure 2J).

### Aberrant bile acid metabolism in patients with DH syndrome based on microbial and serum metabolite functional analysis

The clinical analysis demonstrated that the TBA concentration of patients in the CHB_DH group was significantly higher than those in the CHB_nonDH group, and the trend for the patients with NAFLD was consistent (Table 1). To investigate the underlying biological functions commonly associated with DH syndrome in patients with CHB and NAFLD, we performed functional analyses based on the microbiota and metabolites co-altered in DH syndrome (CHB_DH *vs*. CHB_nonDH and NAFLD_DH *vs*. NAFLD_nonDH, respectively).

**FIGURE 3:**
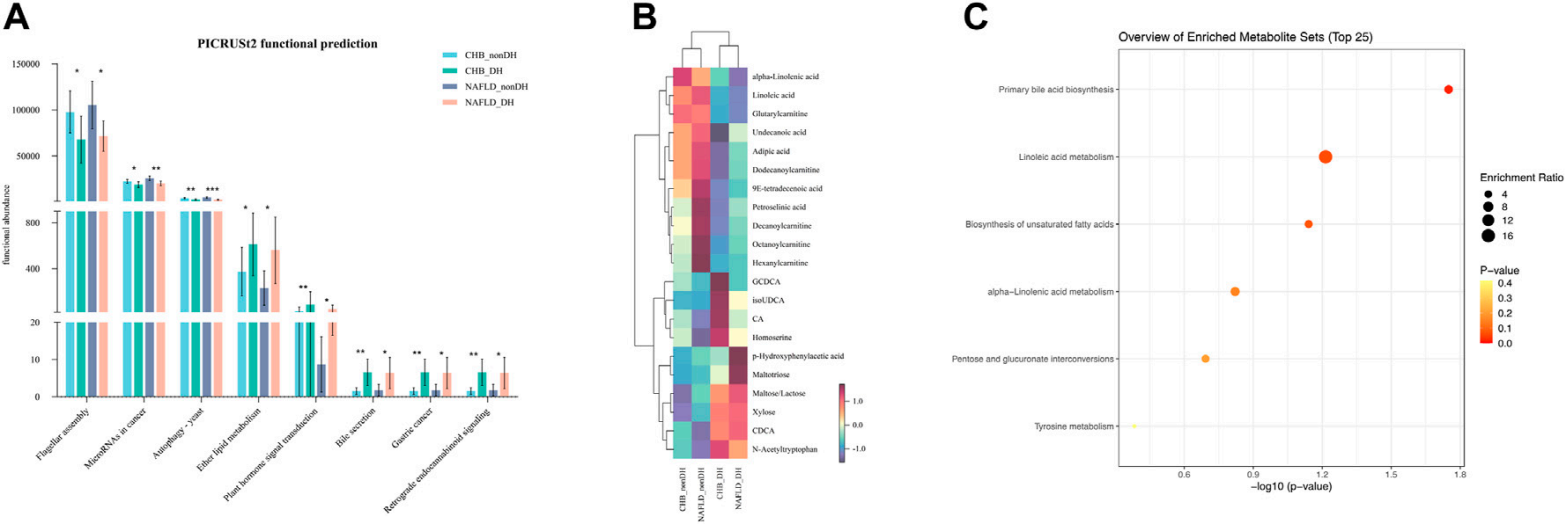
Patients with DH syndrome are associated with aberrant BA metabolism. **(A)** Functional pathways with the same trend predicted based on 870 OTUs, *p < 0.05, **p < 0.01, ***p < 0.001; **(B)** heatmap of 21 metabolites with the same expression trends in DH syndrome groups; **(C)** functional enrichment analysis based on the 21 metabolites.

There were 870 OTUs with the same expression trends for DH syndrome and non_DH syndrome. We used PICRUST2 to predict the biological functions of the foregoing 870 OTUs. Eight pathways were enriched at level-3 KOs with the same variation trend. Compared with the non_DH syndrome groups, flagellar assembly, cancer microRNAs, and autophagy-yeast were decreased in the DH syndrome groups. However, ether lipid metabolism, plant hormone signal transduction, bile secretion, gastric cancer, and retrograde endocannabinoid signaling were predicted increased in the DH syndrome groups (*p* < 0.05) (Figure 3A, Supplementary Table S2).

We also quantified 300 serum metabolites and detected 197 of them. A multivariate control chart of the samples indicated good QC (Supplementary Figure S2A). The most detected metabolites were carbohydrates, organic acids, amino acids, and fatty acids (Supplementary Figure S2B). We used the criterion |Fold change (FC)| > 1.2 to explore the metabolites with the same expression trends between CHB_DH and CHB_nonDH and between NAFLD_DH and NAFLD_nonDH groups. We screened out 21 metabolites. The DH syndrome volunteers had lower serum fatty acids and carnitines and higher serum BAs (glycochenodeoxycholic acid, iso-ursodeoxycholic acid, chenodeoxycholic acid, and cholic acid) and carbohydrates trends than that of patients with the non_DH syndrome (Figure 3B). All 21 metabolites only enriched in the primary BA biosynthesis pathway (*p* < 0.05; Figure 3C).

### Five genera significantly decreased in abundance in patients with DH syndrome

**FIGURE 4:**
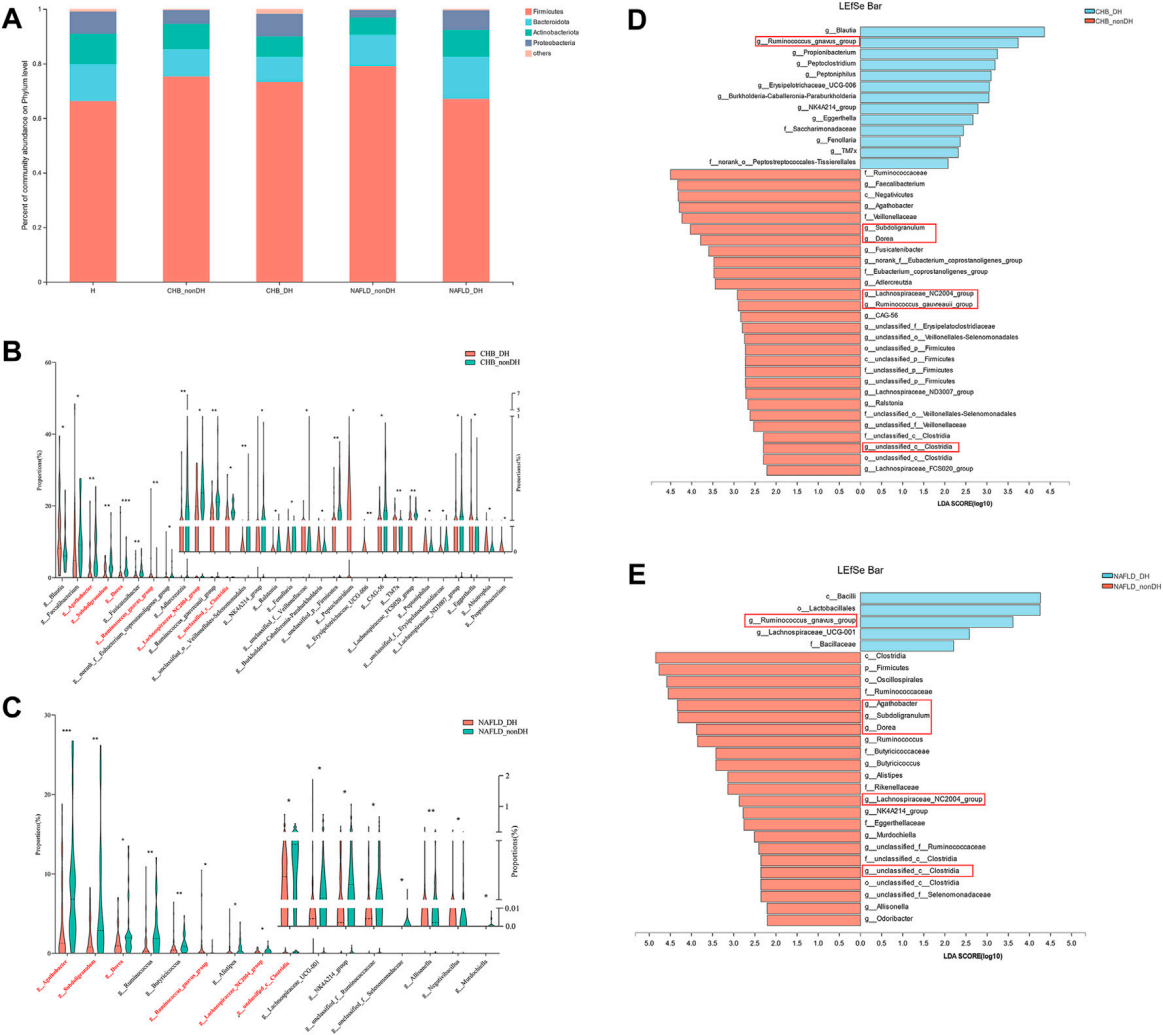
Comparison of the gut microbiome in patients with and without DH syndrome. **(A)** Phylum-level gut microbial composition; **(B)** the relative abundance of 30 different bacterial genera between CHB_DH and CHB_nonDH patients; **(C)** the relative abundance of 16 different bacterial genera between NAFLD_DH and NAFLD_nonDH patients; LEfSe analysis between **(D)** CHB_DH and CHB_nonDH groups; **(E)** NAFLD_DH and NAFLD_nonDH groups.

We then analyzed the compositional differences between the DH syndrome and non_DH syndrome groups at various taxonomic levels. There was no phylum-level compositional difference between the DH and non_DH syndrome groups in patients with CHB or NAFLD (Figure 4A). At the genus level, a total of 30 genera statistically differed between the CHB_DH and CHB_nonDH, and 16 genera differed between the NAFLD_DH and NAFLD_nonDH groups (*p* < 0.05) (Figures 4B,C). Of these, six genera overlapped and had the same trend. We adopted LEfSe to explore the variation of taxonomy in classification. These six genera also significantly differed between CHB and NAFLD patients with and without DH syndrome (*p* < 0.05, LDA score >2.0) (Figures 4D,E).

**FIGURE 5:**
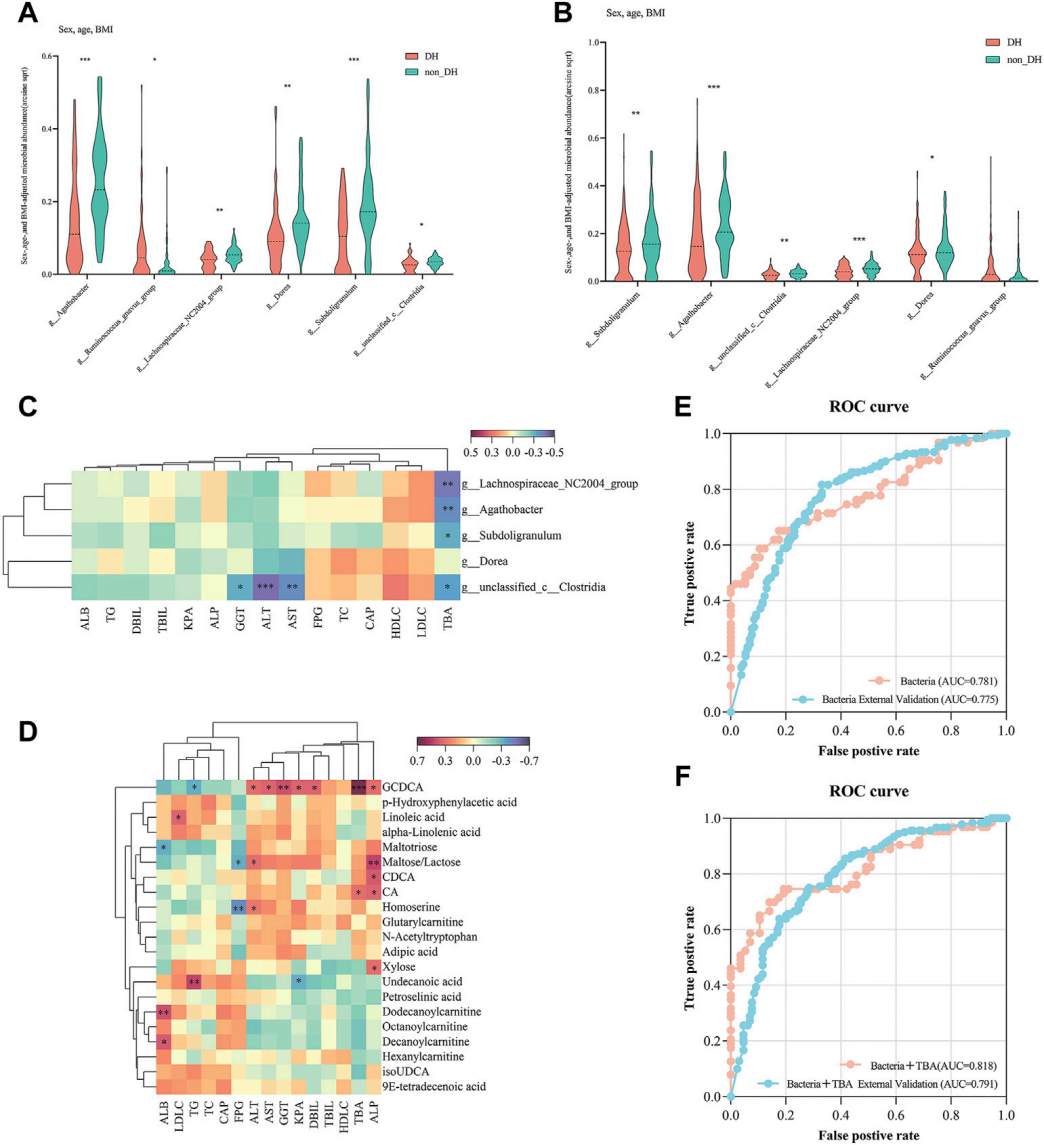
Five genera have diagnostic potential for DH syndrome. Relative expression of six bacterial genera in **(A)** 146 individuals with and without typical DH syndrome and **(B)** all chronic liver disease individuals with and without DH syndrome adjusted for age, sex, and BMI; **(C)** Correlation analysis of DH syndrome-associated gut genera and clinical indices; **(D)** correlation analysis of DH syndrome co-altered trends metabolites and clinical indices; ROC curves were plotted for the diagnosis of DH syndrome in 10-fold cross-validation and test set validation (310 patients with CHB or NAFLD) using the combination of **(E)** five genera, and **(F)** five genera combined with serum TBA. The areas under the ROC curves (AUCs) were calculated.

Compared with the non_DH syndrome group, the relative abundances of the *Agathobacter, Dorea, Lachnospiraceae_NC2004_group, Subdoligranulum,* and *unclassified_c__Clostridia* communities were decreased, while the relative abundance of *Ruminococcus_gnavus_group* was increased in the patients with DH syndrome adjusted for age, sex, and BMI (*p* < 0.05) (Figure 5A). We further analyzed the relative expression of the six genera across all 310 patients with CHB or NAFLD. Except for *Ruminococcus_gnavus_group,* the other five genera showed a consistent statistical difference between DH syndrome and non_DH syndrome groups (Figure 5B). We consider these five genera as DH syndrome-related stool genera. Integrative correlation analysis of the five DH-related genera, serum metabolites, and clinical indicators revealed that the serum TBA concentrations negatively correlated with the abundances of the DH-related genera, four of these genera had statistical significance (*unclassified_c__Clostridia, Subdoligranulum, Agathobacter,* and *Lachnospiraceae_NC2004_group* genera) (Figure 5C). This result indicates patients with DH syndrome lack genera that are negatively correlated with serum TBA concentrations. However, TBA concentrations were significantly positively correlated with the circulating cholic acid (CA) and glycochenodeoxycholic acid (GCDCA) levels. Especially GCDCA was strongly positively correlated with several clinical indices reflecting liver damage (Figure 5D).

### Five genera have diagnostic potential for patients with DH syndrome

We constructed a random forest model to identify the potential utility of these five genera for indicating of DH syndrome according to the relative expression of the five genera in the 146 patients with typical TCM syndromes (Figure 1A, train set). Based on tenfold cross-validation, the model precision for the DH syndrome group was 71%, and the area under the ROC curve (AUC) was 0.781. We then adopted the classifier model to predict DH syndrome in a test set based on all patients with CHB or NAFLD. The classifier precision for DH syndrome was 78.5% with an AUC of 0.775 (Figure 5E, Supplementary Table S3).

In addition, considering the serum TBA levels differed between patients with and without DH syndrome, we also constructed a random forest model combing these five genera with TBA. The addition of TBA to these DH-related stool genera enhanced the diagnostic power with an improved model precision of 79.7% and 78.5% in the internal tenfold cross-validation and the expanded validation, respectively. The related AUC for typical DH syndrome was 0.818, The cut-off value of the model was 0.535, and its true positive rate (TPR) was 0.746. The AUC for DH syndrome in the test set was 0.791 (Figure 5F, Supplementary Table S3).

## Discussion

TCM syndrome is the cornerstone of clinical TCM practice. However, this theory lacks an interpretation from the current biomedical perspective. The present study investigated the characteristics of the gut microbiota and serum metabolites of patients presenting with CHB or NAFLD as well as DH syndrome. We found significant alterations in the microbial community in TCM DH syndrome subjects with different chronic liver diseases such as CHB and NAFLD. DH syndrome is characterized by common alterations in gut microbiota and serum metabolites associated with aberrant bile acid metabolism. Furthermore, we verified five genera as candidate biomarkers for indicating DH syndrome in patients with CHB or NAFLD.

TCM theory believes DH syndrome is clinically common and caused by dysfunction of the spleen and stomach in TCM viscera. Patients with DH syndrome often present with gastrointestinal symptoms such as a bitter taste in the mouth, thirst, halitosis, sticky stool, or constipation (Kang et al., 2015; Zhao et al., 2017). Furthermore, the gut-liver axis has been widely recognized in liver disease, and a healthy intestinal microbial community is vital for maintaining gut and liver homeostasis (Albillos et al., 2020). Thus, there may be some common gut microbiota in the various chronic liver diseases associated with DH syndrome. That is, why we investigated the biological characteristics of DH syndrome for its association with the gut microbiome in the present study.

In TCM syndrome diagnosis, the symptoms have subjective somatosensory differences. The conclusive diagnosis may bias by the experience of the TCM physician. A reliable TCM diagnosis is the premise of follow-up research. Therefore, we employed two rounds of TCM syndrome identification and explored DH syndrome-associated alterations based on the typical participants with consistent TCM diagnosis. The DH syndrome score differed significantly between patients with and without DH syndrome, which indicates the reliability of TCM syndrome diagnosis in the study. We observed significant differences in the changes of gut microbiota according to TCM diagnosis between DH and non_DH syndrome subjects. To further explore the correlation between gut microbiota alteration and DH syndrome, we fitted Gaussian mixture models to the first component of bacterial PLS_DA analysis (Figure 2F; COMP1) and TCM symptomatic PLS_DA analysis (Figure 2G; COMP1), respectively. Both models yielded two normal distributions corresponding to the TCM syndrome groupings. This approach indicates a significant positive correlation between TCM symptoms and intestinal microbiota related to DH syndrome grouping with objective data.

Syndrome diagnosis in TCM and disease diagnosis in Western medicine overlap and complement each other. TCM theory believes that various diseases with the same syndrome may coexist (“Yi Bing Tong Zheng”). Patients with CHB and NAFLD may develop DH syndrome regardless of the difference in pathogenesis between these conditions. Therefore, we sought the intersection of variations with similar trends between the DH and non_DH syndromes in patients with CHB and NAFLD. We found that patients with DH syndrome had relatively higher trends of liver function indices and significantly higher circulating TBA, which was consistent with our previous study. We have retrospectively analyzed the relationships between clinical indices and TCM syndromes in 999 patients with CHB. Patients with CHB and DH syndrome may present with more severe and persistent liver damage than patients without DH syndrome (Liu et al., 2020). Dysregulation of BA metabolism is common in liver disease. Increases in TBA pools are widely observed in metabolic and viral liver diseases and are associated with disease progression (Chavez-Talavera et al., 2017; Sang et al., 2021). BA accumulation in the hepatocytes mediates hepatotoxicity. In this study, patients with DH syndrome presented with elevated trend serum CDCA and GCDCA levels. Treatment of hepatocytes with CDCA upregulates the expression of proinflammatory genes and promotes hepatic inflammation (Allen et al., 2011). GCDCA is synthesized by conjugating CDCA with glycine and induces hepatocyte apoptosis (Vrenken et al., 2008; Cai et al., 2017).

We also found that 870 microbial OTUs and 21 serum metabolites had the same variation trends in both the CHB and NAFLD DH syndrome groups, and the related functional analysis predicted consistent dysregulation of bile acid metabolism. Dysregulation of BA metabolism in patients with DH syndrome aligns with TCM theory. DH syndrome is particularly prominent in TCM jaundice disease. Yin-Chen-Hao decoction is a classical TCM formula for the treatment of DH syndrome. It has been administered in China for ca. 2,000 years and is renowned for its efficacy in regulating bile acid metabolism and treating cholestasis (Cai et al., 2018; Shi et al., 2021). BAs are important bioactive mediators of gut-liver crosstalk (Schneider et al., 2018; Giuliani, 2019; Farooqui et al., 2022), the integrative correlation analysis disclosed that patients with DH syndrome had consistently low abundances of TBA-negative bacterial genera. BAs are directly enzymatically modified by gut bacteria. However, the mechanism associated with gut microbiota and BA metabolism in patients with DH syndrome lacks report and requires further clarification.

In recent years, systematic biology methods have been applied to explore the diagnostic markers of TCM syndromes (Wang and Chen, 2013). For example, an integrated metabonomic and proteomic study on the diagnosis of kidney-yin deficiency syndrome (Jiang et al., 2015). The implementation of metabolomics technology in a large-scale, multi-center urine biomarker identification study on different TCM syndromes (Zhou et al., 2019). Stool microbial profiles have immense potential as diagnostic or prognostic biomarkers for TCM syndromes as the properties of non-invasive and obtainable. A recent study validated two genera in the diagnosis in patients of lung adenocarcinoma-related Qi-Yin deficiency syndrome. However, TCM diagnosis based on gut microbiota is limited and not reported in liver diseases. In this study, we found five significant genera with the same trend in DH and non_DH syndrome groups. *Subdoligranulum, unclassified_c__Clostridia, Agathobacter,* and *Dorea* are implicated in short-chain fatty acid (SCFA) biosynthesis (Jiao et al., 2018; Che et al., 2019; Liu et al., 2019; Lloyd-Price et al., 2019), SCFAs maintain intestinal barrier function and are anti-inflammatory (Maslowski et al., 2009; Macia et al., 2015). A decrease in the relative abundance of *Subdoligranulum* positively correlated with intestinal inflammation (Kim et al., 2021). Hence, patients with DH syndrome are prone to this condition. We explored and verified the diagnostic potential of these five genera for DH syndrome. The AUC of the random forest-based classifier for DH syndrome was 0.781 and 0.775 in the 10-fold cross-validation and test set validation, respectively. To improve the diagnostic efficiency, we combined these five genera with serum TBA, the related diagnostic AUCs for DH syndrome were 0.818 and 0.791, respectively. Therefore, these bacterial genera may be candidates for the diagnosis of TCM DH syndrome. The combination of gut microbiota and serum TBA may be a better diagnostic tool for diagnosing DH syndrome in subjects with chronic liver diseases such as CHB and NAFLD.

We acknowledge several limitations of this study. First, we found increased serum TBA expression and aberrant BA metabolism in individuals with DH syndrome with CHB or NAFLD. However, the causality of DH-associated microbiota, metabolites, and BA metabolism remains unclear. Second, we explored common changes in gut microbiota according to TCM diagnosis in patients with DH syndrome. We validated five genera with diagnostic potential for DH syndrome in patients with chronic liver disease. However, the external test set and training set have overlap (38.7% of the test set). Given the possible overfitting error of the random forest model, future research should replicate this study using a larger sample size of individuals with DH syndrome. Third, the gut microbiota in this study was assessed by 16S rRNA gene sequencing, lacking the quantitative range of these five genera. We hope to modify the diagnostic model with more accurate values in the future, such as Shotgun metagenomic sequencing data. DH syndrome is not common in healthy individuals. In this study, none of the healthy individuals was diagnosed with typical DH syndrome according to the same criteria in patients with CHB or NAFLD. Therefore, the changes in gut microbiota and serum TBA in healthy volunteers with and without DH syndrome cannot be determined yet. We hope to observe the correlated variation in healthy volunteers in larger sample data in the future.

## Conclusion

This study addresses common microbial signatures of TCM DH syndrome in patients with CHB or NAFLD. We explored and validated five DH-specific genera combined with serum TBA as a non-invasive tool for diagnosing DH syndrome in patients with chronic liver disease. The results of this study contribute to the understanding and diagnosis of DH syndrome based on gut microbiota.

## Data Availability

The datasets presented in this study can be found in online repositories. The names of the repository/repositories and accession number(s) can be found below: NCBI BioProject, PRJNA809132.
