# Supplementary material for: Distinct common signatures of gut microbiota associated with damp-heat syndrome in patients with different chronic liver diseases
Source: Front Pharmacol. 2022 Nov 17;13:1027628. doi: 10.3389/fphar.2022.1027628 (PMC9712756; doi:10.3389/fphar.2022.1027628)
Supplement: Supplementary file 1 [file Table1.DOCX]

**Supplementary Table 1.** Dietary intake comparation between groups

|  | H (n=26) | CHB_DH (n=21) | CHB_nonDH (n=29) | *P* value^a^ | NAFLD_DH (n=42) | NAFLD_nonDH (n=28) | *P* value^b^ |
| --- | --- | --- | --- | --- | --- | --- | --- |
| Fish (including marine and freshwater fish) | 7.69 | 9.52 | 17.24 | 0.716 | 23.81 | 14.29 | 0.329 |
| Shrimp, Crab, Shellfish | 15.38 | 4.76 | 6.90 | 1 | 14.29 | 0.00 | 0.098 |
| Pickled aquatic products | 0.00 | 4.76 | 3.45 | 1 | 9.52 | 3.57 | 0.636 |
| Tea | 7.69 | 14.29 | 17.24 | 1 | 28.57 | 35.71 | 0.528 |
| Liquor | 0.00 | 0.00 | 0.00 | NS | 7.14 | 0.00 | 0.399 |

a: CHB_DH vs. CHB_nonDH; b: NAFLD_DH vs. NAFLD_nonDH.
