# Supplementary figures and images for: Distinct common signatures of gut microbiota associated with damp-heat syndrome in patients with different chronic liver diseases
Source: Front Pharmacol. 2022 Nov 17;13:1027628. doi: 10.3389/fphar.2022.1027628 (PMC9712756; doi:10.3389/fphar.2022.1027628)

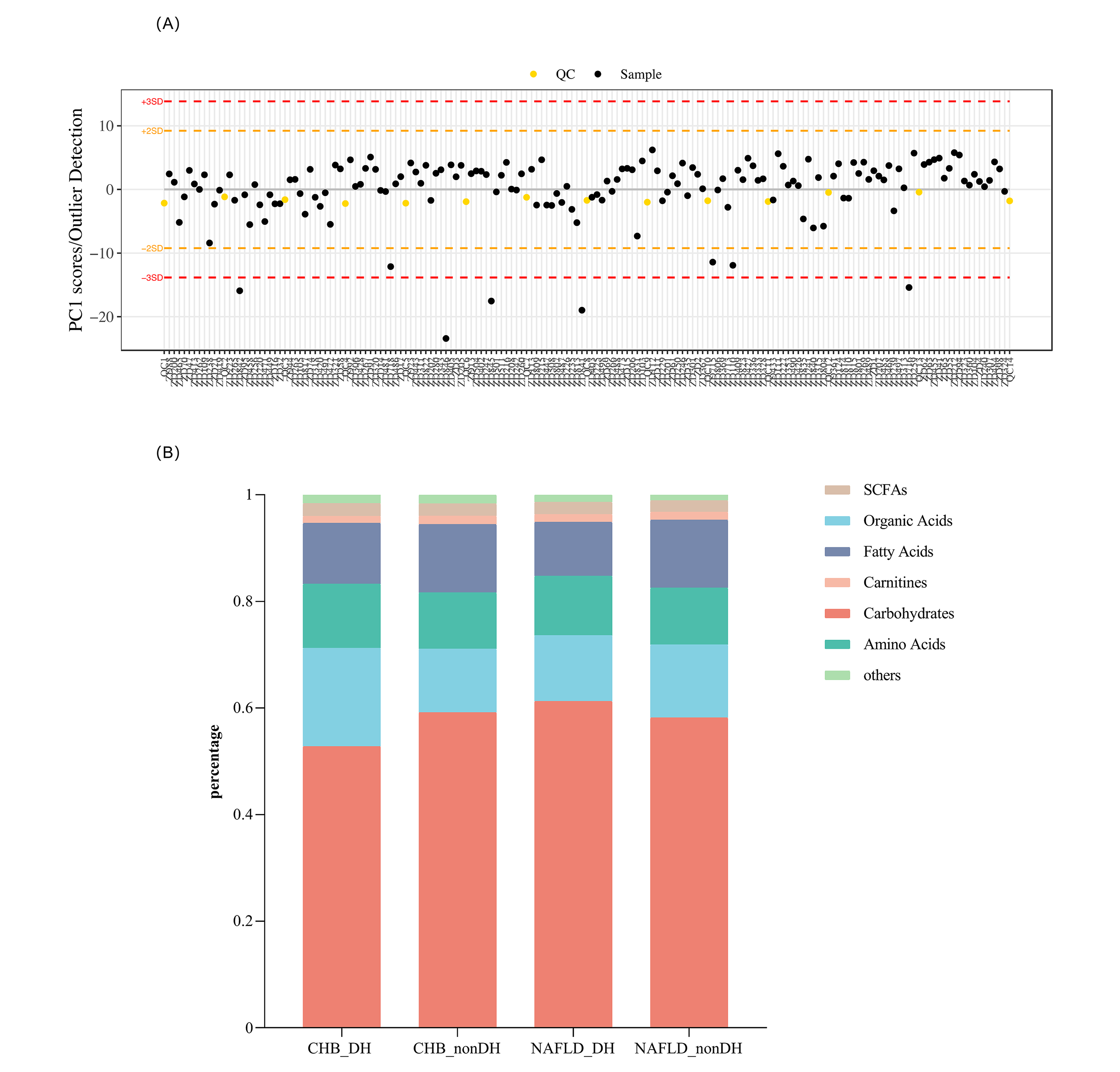

Supplement: Supplementary file 2 [file Image2.TIF]

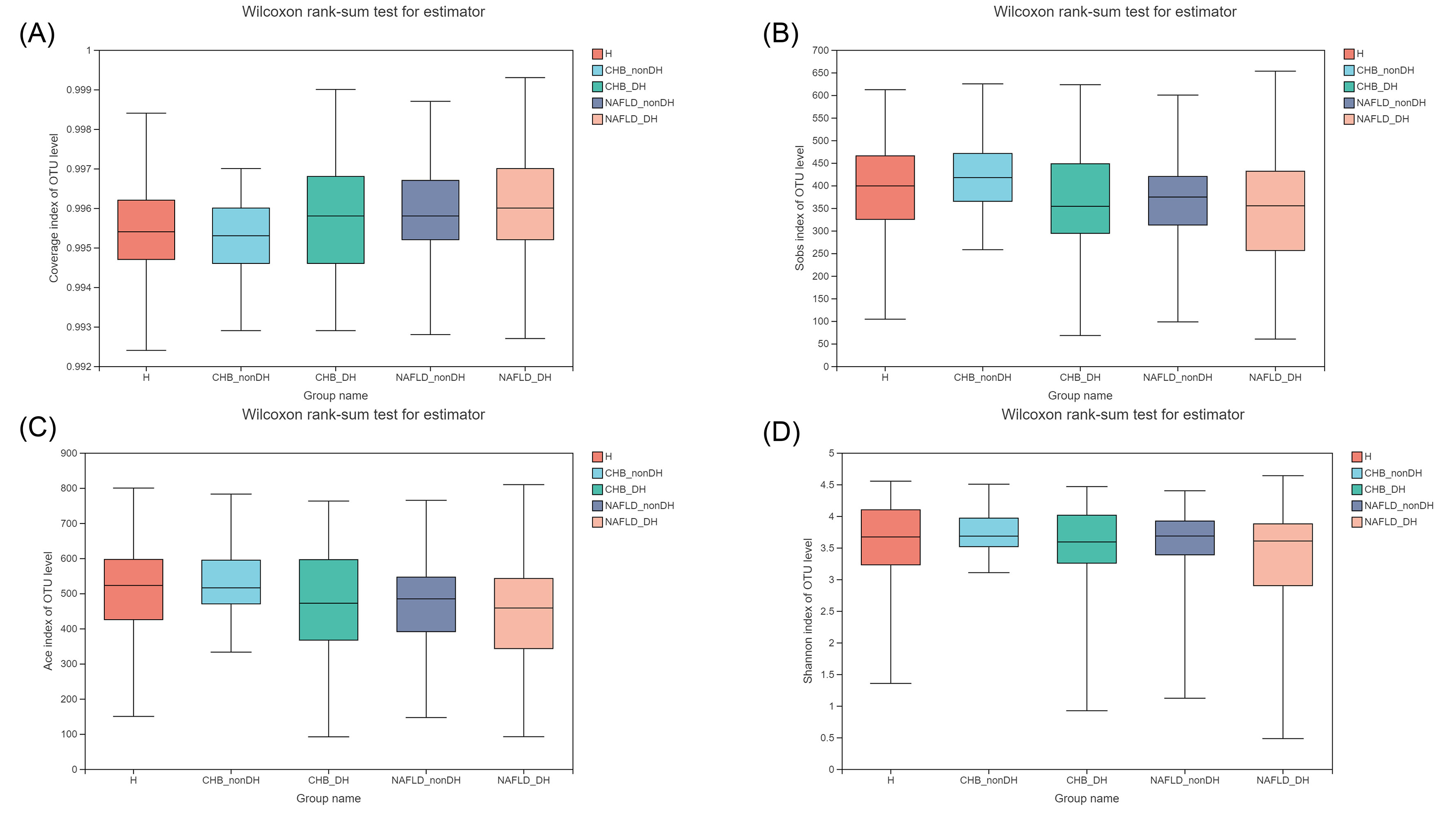

Supplement: Supplementary file 3 [file Image1.TIF]
