# Supplementary material for: Distinct common signatures of gut microbiota associated with damp-heat syndrome in patients with different chronic liver diseases
Source: Front Pharmacol. 2022 Nov 17;13:1027628. doi: 10.3389/fphar.2022.1027628 (PMC9712756; doi:10.3389/fphar.2022.1027628)
Supplement: Supplementary file 4 [file Table2.DOCX]

**Supplementary Table 2.** Eight pathways were enriched at level-3 KOs with the same variation trend predicted based on 870 OTUs.

|  | CHB_DH  (*n* = 21) | CHB_nonDH  ( *n* = 9) | *P* value^a^ | NAFLD_DH  ( *n* = 42) | NAFLD_nonDH  ( *n* = 28) | *P* value^b^ |
| --- | --- | --- | --- | --- | --- | --- |
| Flagellar assembly | 50008.1(65438.32) | 83661.74(82406.96) | 0.042 | 53940.88(63985.1) | 93737.63(75276.82) | 0.019 |
| Gastric cancer | 5(8.5) | 1(1.99) | 0.003 | 1(5.99) | 0.01(0.99) | 0.044 |
| MicroRNAs in cancer | 18910.93(5760.03) | 22511.26(6593.12) | 0.035 | 21650.32(10471.87) | 25007.1(8719.57) | 0.004 |
| Plant hormone signal transduction | 17(40.2) | 0.4(4.69) | 0.007 | 5.2(40.25) | 0.4(5.04) | 0.035 |
| Bile secretion | 5(8.5) | 1(1.99) | 0.003 | 1(5.99) | 0.01(0.99) | 0.044 |
| Autophagy - yeast | 2219.2(1919.7) | 4304.71(2929.92) | 0.003 | 2379.55(2280.18) | 4635.12(3131.09) | ＜0.001 |
| Ether lipid metabolism | 467.78(735.49) | 129.85(435.93) | 0.046 | 169.31(526.97) | 92.54(150.35) | 0.045 |

The data was expressed as median (interquartile range); Mann-Whitney *U* test was used to analyze significant differences between groups. a: CHB_DH vs. CHB_nonDH; b: NAFLD_DH vs. NAFLD_nonDH.
