# Supplementary material for: Distinct common signatures of gut microbiota associated with damp-heat syndrome in patients with different chronic liver diseases
Source: Front Pharmacol. 2022 Nov 17;13:1027628. doi: 10.3389/fphar.2022.1027628 (PMC9712756; doi:10.3389/fphar.2022.1027628)
Supplement: Supplementary file 5 [file Table3.DOCX]

| Model | | TP Rate | FP Rate | Precision | Recall | F-Measure | MCC | ROC Area | PRC Area |
| --- | --- | --- | --- | --- | --- | --- | --- | --- | --- |
| Five genera | Tenfold cross-validation | 0.698 | 0.316 | 0.71 | 0.698 | 0.704 | 0.382 | 0.781 | 0.842 |
|  | Test set Validation (310 patients) | 0.711 | 0.269 | 0.785 | 0.711 | 0.746 | 0.437 | 0.775 | 0.791 |
| Five genera + TBA | Tenfold cross-validation | 0.746 | 0.211 | 0.797 | 0.746 | 0.77 | 0.535 | 0.818 | 0.869 |
|  | Test set Validation (310 patients) | 0.75 | 0.285 | 0.785 | 0.75 | 0.767 | 0.462 | 0.791 | 0.809 |

**Supplementary Table 3.** Detailed accuracy by class
